# Co-culture Wood Block Decay Test with Bacteria and Wood Rotting Fungi to Analyse Synergism/Antagonism during Wood Degradation

**DOI:** 10.21769/BioProtoc.4837

**Published:** 2023-10-05

**Authors:** Julia Embacher, Susanne Zeilinger, Sigrid Neuhauser, Martin Kirchmair

**Affiliations:** Department of Microbiology, University of Innsbruck, Innsbruck, Austria

**Keywords:** ENV 12038 standard, Wood decay, Bacterial–fungal interactions, Synergism/Antagonism, *Serpula lacrymans*

## Abstract

Mixed communities of fungi and bacteria have been shown to be more efficient in degrading wood than fungi alone. Some standardised protocols for quantification of the wood decay ability of fungi have been developed (e.g., DIN V ENV 12038:2002 as the legal standard to test for the resistance of wood against wood-destroying basidiomycetes in Germany). Here, we describe a step-by-step protocol developed from the official standard DIN V ENV12038 to test combinations of bacteria and fungi for their combined wood degradation ability. Equally sized wood blocks are inoculated with wood decay fungi and bacterial strains. Axenic controls allow the analysis of varying degradation rates via comparison of the wood dry weights at the end of the experiments. This protocol provides new opportunities in exploration of inter- and intra-kingdom interactions in the wood-related environment and forms the basis for microcosm experiments.

Key features

• Quantification of wood decay ability of mixed cultures.

• Allows testing if fungi are more efficient in degrading wood when bacteria are present.

## Background

In nature, basidiomycetous fungi are associated with other microbes including prokaryotes and eukaryotes. This results in complex competitive and antagonistic interactions as well as commensal and mutualistic behaviour (Boer et al., 2005; Kobayashi and Crouch, 2009). Fungi are the most efficient wood decomposers, as their multicellular appearance and hyphal growth harbour a mobility advantage in comparison to prokaryotes. Nonetheless, bacteria are known to have direct influence on the decay process as well, by e.g., degrading complex wood components like cellulose, lignin, and hemicellulose (McGuire and Treseder, 2010) or by altering wood permeability and structure, thus improving accessibility of the wooden microfibrils (Clausen, 1996), aiding other organisms in wood decay. Synergistically acting species benefit from each other’s enzymatic abilities when they are cultivated on wood (Cortes-Tolalpa et al., 2017; Sugano et al., 2021). These results highlight the importance of inter- and intra-kingdom interactions, as they show that the combined action of different species enhances the decay ability of the whole community.

To date, several protocols and studies exist for the assessment of wood resistance against wood-destroying basidiomycetes [e.g., DIN V ENV 12038:2002 (German Institute for Standardisation, 2002), wood block test of Bravery (1978), or work of Lohwag (1965) and Hegarty et al. (1987)]. However, as these protocols evolved in large part to study timber preservation and were not designed to study the influence of other microbes, we adapted a protocol based on DIN V ENV 12038:2002 to the here described co-culture wood block decay test. This step-by-step protocol provides an easily feasible standard procedure for the assessment of wood decay properties of mixed cultures in comparison to axenic decay rates by evaluating the dry weight of wood blocks that were exposed to microbial deterioration. The purpose of this wood decay assay is to determine the influence of bacteria on the decay properties of wood rotting fungi like *Serpula lacrymans* [see e.g., Embacher et al. (2022), article in prep.].

## Materials and reagents

**Fungal and bacterial strains**

1. Fungal strain to test [*Serpula lacrymans*, origin: Innsbruck, no. 1SLIBK2018 (Embacher et al., 2021)]
2. Bacterial strains to test [e.g., *Microbacterium* spp. (Embacher et al., 2021 and 2022)]

**Materials**

1. Autoclaved wood blocks (50 mm × 25 mm × 15 mm) (e.g., *Picea abies*; all blocks for one experiment should originate from the same batch timber)
2. Vessels (88 mm height, Ø 75 mm, volume 300 mL, e.g., ROTILABO^®^, catalog number: EP28.1) filled with 25 mL malt extract agar
3. Petri dishes 94 mm × 16 mm, without vents (Greiner Bio-One, catalog number: 632180)
4. Pre-cultured bacterial isolates to be tested
5. Pre-cultured fungal strain(s) to be tested (e.g., the wood rotting fungus *S. lacrymans*)
6. Fisherbrand^TM^ Easy Reader^TM^ conic centrifuge tubes 15 mL, PP (Fisher Scientific, catalog number: 11819650)
7. Sterile toothpicks
8. 1.5 mL microcentrifuge tubes (Greiner Bio-One, catalog number: 616201)

**Reagents**

1. Casein peptone (Roth, catalog number: 8952.2)
2. Soy peptone (Roth, catalog number: 2365.2)
3. NaCl (Roth, catalog number: 0601.1)
4. Agar (Roth, catalog number: 5210.2)
5. Malt extract (Roth, catalog number: X976.2)
6. dH_2_O
7. D(+)-Glucose monohydrate (Roth, catalog number: 6780.1)
8. (NH_4_)_2_HPO_4_ (Roth, catalog number: P736.1)
9. KH_2_PO_4_ (Roth, catalog number: 3904.1)
10. MgSO_4_·7H_2_O (Roth, catalog number: T888.1)
11. CaCl_2_·2H_2_O (Roth, catalog number: T885.1)
12. FeCl_3_ (Roth, catalog number: 5192.1)
13. Thiamine-HCl (Roth, catalog number: T911.1)
14. EDTA disodium salt (Roth, catalog number: 8043.1)
15. ZnSO_4_·7H_2_O (Roth, catalog number: K301.2)
16. H_3_BO_3_ (Roth, catalog number: 5935.1)
17. MnCl_2_·4H_2_O (Roth, catalog number: 0276.1)
18. CoCl_2_·6H_2_O (Roth, catalog number: 7095.1)
19. CuSO_4_·5H_2_O (Roth, catalog number: 8175.6)
20. (NH_4_)_6_Mo_7_O_24_·4H_2_O (Roth, catalog number: 7311.1)
21. FeSO_4_·7H_2_O (Roth, catalog number: P015.1)
22. KOH (Roth, catalog number: 6751.1)
23. NaOH (9356.1)
24. PCR reagents:
  - Red Taq 2× DNA Polymerase Master Mix (VWR, Radnor, USA)
  - Primers 27F (5′-AGA GTT TGA TCA TGG CTC A-3′) and 1492R (5′-TAC GGT TAC CTT GTT ACG ACT T-3′) (both 10 μM)
  - Distilled water
  - Bovine serum albumin (BSA) 2% [(Roth, catalog number: 3854.2); see as well (Embacher et al., 2021)]
25. 1% agarose gel for gel electrophoresis system [e.g., agarose powder (Sigma, catalog number: A9539)]
26. Polyethylene glycol 6000 (PEG) 20% (Roth, catalog number: 0158.1)
27. 80% EtOH (-20 °C)
28. Nuclease-free water
29. Tryptone soya agar (TSA) plates (see Recipes)
30. TSA soft agar (freshly prepared on harvesting day, store at ~50 °C until usage) (see Recipes)
31. Liquid Tryptone soya (TS) medium (see Recipes)
32. Malt extract agar (MEA) (see Recipes)
33. Modified Melin-Norkrans (MMN) (see Recipes)
34. Hutner’s Trace metals (see Recipes)
35. 0.85% NaCl solution (see Recipes)
36. Sodium borate buffer (20× Stock solution, pH 8) (for agarose gel electrophoresis, see Recipes)

**Recipes**

1. **Tryptone soya agar (TSA) plates**
  Casein peptone 1.5% (w/v), 15 g
  Soy peptone 0.5% (w/v), 5 g
  NaCl 0.5% (w/v), 5 g
  Agar 1.8% (w/v), 18 g
  dH_2_O, add up to 1 L
  (pH = 7.3 ± 0.2)
  Prepare TSA medium, autoclave (120 °C), let it cool down, pour into Petri dishes, and let cool to room temperature (RT). Store at 4 °C.
2. **TSA soft agar**
  Casein peptone 1.5% (w/v), 15 g
  Soy peptone 0.5% (w/v), 5 g
  NaCl 0.5% (w/v), 5 g
  Agar 0.7% (w/v), 7 g
  dH_2_O, add up to 1 L
  (pH = 7.3 ± 0.2)
  Prepare TSA medium and autoclave (120 °C). Store at ~50 °C until usage for plate casting after Koch.
3. **Liquid Tryptone soya TS medium**
  Casein peptone 1.5% (w/v), 15 g
  Soy peptone 0.5% (w/v), 5 g
  NaCl 0.5% (w/v), 5 g
  dH_2_O, add up to 1 L
  (pH = 7.3 ± 0.2)
  Prepare TS liquid medium, autoclave (120 °C), and let it cool down. Store at 4 °C.
4. **Malt extract agar (MEA)**
  Malt extract 3% (w/v), 30 g
  Soy peptone 0.3% (w/v), 3 g
  Agar 1.8% (w/v), 18 g
  dH_2_O, add up to 1 L
  (pH = 3.5 ± 0.2)
  Prepare MEA medium, autoclave (120 °C), and cool it down to 50 °C. Pour into Petri dishes and let cool to RT. Store at 4 °C.
5. **Modified Melin-Norkrans (MMN) [after Tauber et al. (2016)]**
  Glucose, 5 g
  (NH_4_)_2_HPO_4_, 0.25 g
  KH_2_PO_4_, 0.5 g
  MgSO_4_·7H_2_O, 0.15 g
  CaCl_2_·2H_2_O, 0.067 g
  NaCl, 0.025 g
  FeCl_3_ (1%), 1.2 mL
  Thiamine-HCl (10%), 1 μL
  Hutner’s trace metals (0.01×), 100 μL
  Agar, 20 g
  dH_2_O, add up to 1 L
  (pH = 5.6)
  Prepare FeCl_3_, Thiamine-HCl (10%), and Hutner’s trace metals (0.01×) (Hutner et al., 1950) separately. Add them to the media while mixing on the rotary shaker. Measure the pH (and adjust if necessary) and adjust the media to 1,000 mL. Autoclave at 120 °C, let it cool down, pour into Petri dishes, and let cool to RT. Store at 4 °C.
6. **Hutner’s trace metals**
  EDTA disodium salt, 50 g in 250 mL dH_2_O
  ZnSO_4_·7H_2_O, 22 g in 100 mL
  H_3_BO_3_, 11.4 g in 200 mL
  MnCl_2_·4H_2_O, 5.06 g in 50 mL
  CoCl_2_·6H_2_O, 1.61 g in 50 mL
  CuSO_4_·5H_2_O, 1.57 g in 50 mL
  (NH_4_)_6_Mo_7_O_24_·4H_2_O, 1.10 g in 50 mL
  FeSO_4_·7H_2_O, 4.99 g in 50 mL
  20% KOH solution (w/v)
  For 1 L final mix, dissolve each component in the volume of water indicated. The EDTA should be dissolved in boiling water, and the FeSO_4_·7H_2_O should be prepared last to avoid oxidation.
  Mix all solutions except EDTA. Bring to boil, then add the EDTA solution. The colour of the mixture turns to green. When everything is dissolved, let cool to 70 °C. While keeping the temperature at 70 °C, add 85 mL of hot KOH (20%). Cool to RT and fill up to 1 L final volume. Close the flask with a cotton plug (allows air exchange) and swirl it once a day while incubating for 1–2 weeks. Usually, the solution will initially be clear green but turns dark red or purple over the next few days, leaving a rust-brown precipitate. If no precipitate forms or the solution remains green, check the pH (should be at approximately 6.7; if there is a big deviation, try adding either KOH or HCl to adjust it).
  Filter through two layers of Whatman #1 filter paper and repeat, if necessary, until the solution is clear. Store refrigerated or frozen in convenient aliquots.
7. **0.85% NaCl Solution**
  Sodium chloride 0.85% (w/v), 8.5 g
  dH_2_O, add up to 1 L
  Prepare solution, autoclave (120 °C), and let cool down. Store at RT.
8. **Sodium borate buffer (20× stock solution, pH = 8)**
  Boric acid, 48 g (concentration 1 M)
  NaOH pellets, 8 g
  dH_2_O, add up to 1 L
  Prepare 900 mL of dH_2_O in a suitable container and add the boric acid and the sodium hydroxide to the solution. Stir until all solids are dissolved. Adjust pH to 8 by adding boric acid or NaOH. Fill to 1 L with dH_2_O.
  Dilute the 20× stock solution to 1× before usage for gel electrophoresis (can be used for preparation for agarose gels and in the gel tank).

## Equipment

1. Eppendorf centrifuge 5810 equipped with A-4-81 rotor (Eppendorf SE, Hamburg, Germany)
2. Heraeus Fresco^TM^ 17 microcentrifuge equipped with 24 × 1.5/2.0 mL rotor with ClickSeal^TM^ biocontainment lid (Thermo Scientific, Waltham, Massachusetts, USA)
3. Counting chamber Thoma (depth: 0.01 mm, 0.0025 mm^2^, Assistent, Glaswarenfabrik Karl Hecht, Sondheim vor der Rhön, Germany)
4. Autoclave (HMC Europe, HICLAVE, catalog number: HGS-133)
5. Incubator (temperature range: 10–30 °C, e.g., Heraeus Vötsch, Vienna, Austria)
6. Benchtop orbital incubator shaker (New Brunswick Scientific, Edison, model: Innova^®^ 40/40R)
7. Chemically resistant diaphragm vacuum pump model N810 FT.18 (KNF LABOPORT, Hamburg, Germany)
8. Vacuum filter manifold and sterile filtration funnels (Millipore Sigma^TM^, Microfi^l®^, Merck Millipore Billerica, USA)
9. Nitrocellulose filter (0.45 μm; diam. 47 mm, part no: 1215230, GVS North America, Sanford, USA)
10. Overhead shaker model REAX2 (Heidolph Instruments, type: 541-21001-00)
11. Heating oven, 3.8 cu ft, 230 VAC (Memmert, UNB500/230 Basic Oven)
12. Balance (PM4600 DeltaRange^®^, Mettler-Toledo, Vienna, Austria)
13. Microbiological safety cabinet (Platinum SF, Kojair Tech Oy, Mänttä-Vilppula, Finland)
14. Carton package (to guarantee incubation in darkness)
15. Sterile glass Petri dishes (100 mm × 20 mm Rotilabo^®^, Carl Roth, Karlsruhe, Germany)
16. Sterile plier
17. Sterilizable punch (that gives e.g., plugs with ~0.8 cm × 0.7 cm × 1.1 cm)
18. Tweezers
19. PCR cycler (Primus 96 advanced, Peqlab Biotechnologie, Erlangen, Germany)
20. Horizontal gel electrophoresis system (RunOne^TM^ Electrophoresis Cell, EmBi Tech, San Diego, USA)
21. Gel Doc^TM^ EZ Imager 30016395-0000 171800 (Bio-Rad Laboratories, Hercules, California, USA)
22. Thermomixer^®^ comfort 5355 (Eppendorf SE, Hamburg, Germany)

## Software

1. Microsoft Excel version 2209
2. RStudio version 4.0.3 (2020-10-10)

## Procedure

- **Pre-experiments to determine bacterial viability on wood blocks**
  1. Grow bacterial isolates in 5 mL of TS liquid medium overnight (25 °C, 220 rpm, uncontrolled light/dark conditions).
  2. Harvest bacteria and wash them with 1 mL of 0.85% NaCl solution (3×, centrifugation at 5,900× *g* for 10 min).
  3. Use a Thoma chamber to adjust the bacterial suspension to 10^8^ CFU/mL with 0.85% NaCl solution (end volume minimum 10 mL).
  4. Immerse the autoclaved wood blocks with bacterial suspension and incubate them in a sterile glass Petri dish overnight at 25 °C.
  5. The next day, imprint the wood blocks gently on TSA plates, using a sterilised tweezer. Remove the wood blocks. Incubate at 25 °C overnight to 24 h.
  6. If bacteria grow rapidly on TSA plates, they are useable for the main experiment.
- **Preparation of the main experiment**
  1. Establish fungus on solid agar medium (Figure 1; here, *S. lacrymans* was pre-cultivated on modified Melin-Norkrans (MMN) for 3–4 weeks at 25 °C).
    **Figure 1.**
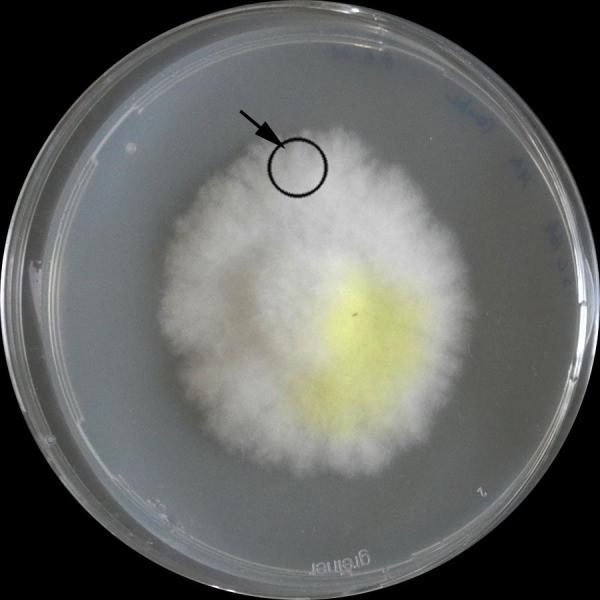
Established culture of *S. lacrymans*. Arrow: peripheral zone
  2. Add mycelia-covered agar plugs (use a sterilizable punch, ~0.8 cm × 0.7 cm × 1.1 cm) to vessels filled with 25 mL of MEA medium.
*Note: Use the peripheral zone of the pre-culture(s). Set aside some vessels, as they serve as control (Figure 1).*
  3. Incubate in darkness for four weeks (25 °C).
    In the meantime:
  4. Number the wood blocks (50 ± 0.5 mm × 25 ± 0.5 mm × 15 ± 0.5 mm, e.g., from *Picea abies*).
  5. Autoclave wood blocks (120 °C) and note their weights (maintain sterile conditions, e.g., put balance under the sterile workbench). Keep them in a sterile environment until use.
    - Put a minimum of four moisture control specimen wood blocks in the oven and dry them at ~50 °C for 72 h.
    - Weigh and record the dry mass of the moisture control wood blocks.
  6. Number and weigh glass Petri dishes.
- **Setup of the main experiment**
  1. Start to prepare bacteria approximately 2–3 days before fungal cultures are ready. Culture them on TSA medium (25 °C).
  2. Inoculate 5 mL of liquid TS medium with bacteria (25 °C, 220 rpm, overnight).
  3. Harvest bacteria and wash them with 0.85% NaCl solution (3×, centrifugation at 5,900× *g* for 10 min)
  4. Use a Thoma chamber to adjust the bacterial suspension to 10^8^ CFU/mL (use 0.85% NaCl solution).
  5. Immerse the numbered, autoclaved, and weighed wood blocks with the bacterial suspension, remove them immediately, drain the excess suspension (sterilised filter paper) and put one (immersed) wood block in each vessel with fungal mycelium. Prepare a minimum of five replicates.
*Note: Set aside some vessels as they serve as control.*
  6. For each approach, put wood blocks in vessels filled with MEA but without mycelium, as these are additional controls (axenic bacterial controls) (minimum triplicates per bacterium).
  7. Treat a minimum of 14 wood blocks solely with 0.85% NaCl solution and place them on top of the mycelium (axenic fungal control).
  8. Incubate the vessels at 25 °C in darkness (e.g., put them in a carton package) for eight weeks (Figure 2).
    **Figure 2.**
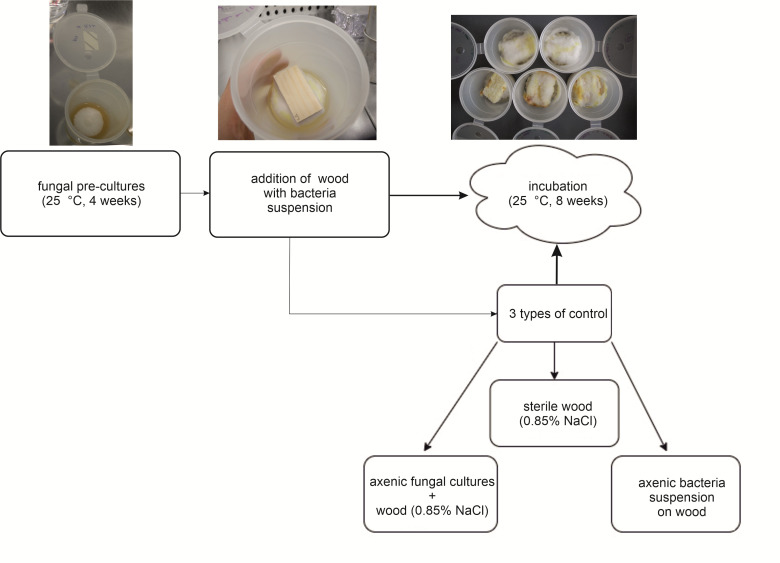
Preparation and setup procedure of the wood decay experiment. Illustration of sections B and C.
- **Harvesting**
  1. Remove superficial mycelium from the wood blocks with sterilised tweezers (Figure 2). Collect the mycelium under sterile conditions for further use in step D3.
  2. Imprint the wood blocks on TSA plates and incubate them at 25 °C.
    - Put each wood in one numbered and pre-weighed glass Petri dish; the numeration should match.
    - Weigh the wood blocks + Petri dish.
    - Dry the wood blocks at approximately 50 °C.
  3. Weigh the mycelium collected from the wood.
  4. Dilute the mycelium sample 1:10 with NaCl solution (0.85%).
  5. Shake the mycelium solution for 30 min on a rotary shaker.
  6. Plate 100 μL of the solution on TSA agar plates and incubate at 25 °C.
  7. Use the residual solution.
    - For plate casting after Koch (if less than 1 mL):
      i. Mix residual solution with 3 mL of TSA soft agar.
      ii. Vortex gently.
      iii. Pour evenly on TSA plates.
    - For concentration on a nitrocellulose filter with vacuum (if more than 1 mL):
      i. Clean the filter unit with 96% EtOH and sterilise the tweezers.
      ii. Install the sterile filtration funnel and the filter.
      iii. Pour the solution on the filter.
      iv. Apply vacuum.
      v. Put the filter on TSA medium.
  8. Incubate all agar plates at 25 °C and check daily for growth (Figure 3).
    **Figure 3.**
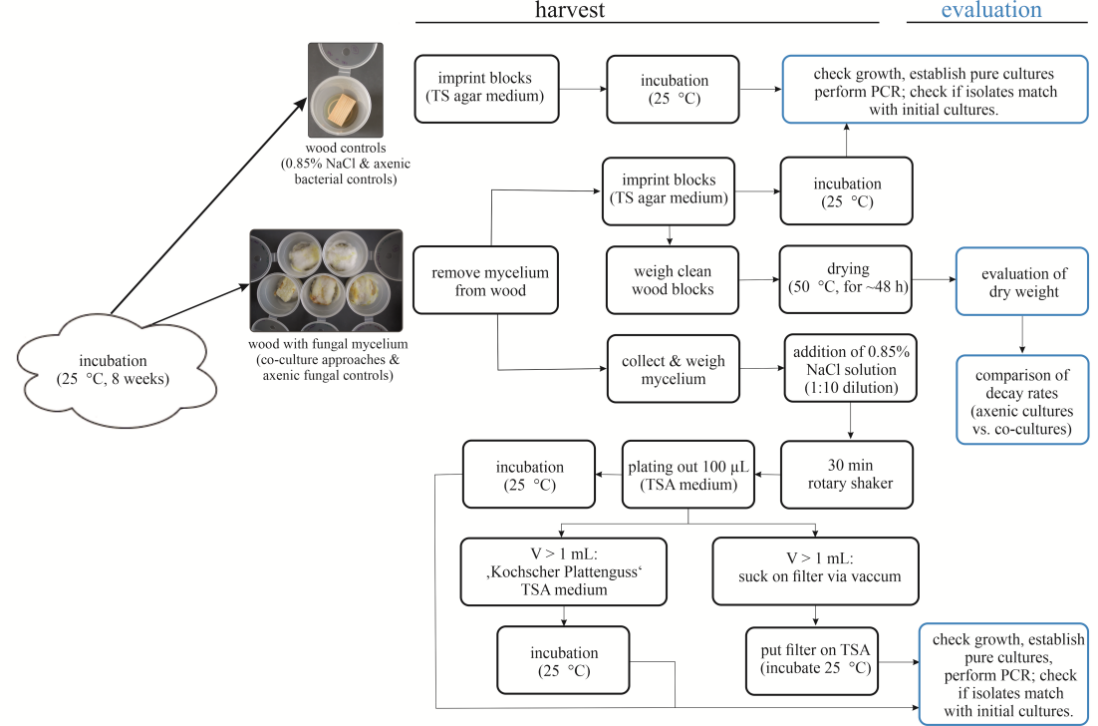
Harvesting and evaluation procedure of the wood-decay experiment. Illustration of sections D and E.
- **Evaluation**
  1. Weigh dried wood blocks.
    - The wood blocks can stay in the glass Petri dish, as the weight was determined at the start of the experiment.
    - Weigh them daily until they are dry (should take no longer than 2–3 days).
  2. Check TSA plates for growth, documenting on which plates growth occurs.
    - Use single bacteria colonies for establishment of pure cultures (TSA agar plate, 25 °C).
    - Perform colony PCR and check if bacteria match with applied species by sequencing the 16S rRNA gene (Embacher et al., 2021).
      i. Prepare PCR reaction: mix 12.5 μL of Red Taq DNA Polymerase Master Mix (2×) with 0.625 mL of each of the primers 27F and 1492R (10 μmol), 10.75 μL of distilled water, and 0.5 μL of BSA (2%) (total volume = 25 μL).
      ii. Pick a single bacterial colony not older than three days with a sterile toothpick and add it to the PCR mixture.
      iii. Transfer the reactions to the PCR cycler: PCR conditions are 95 °C for 10 min, 30 cycles of 95 °C for 30 s, 53 °C for 30 s, 72 °C for 45 s, and a final elongation step at 72 °C for 10 min.
      iv. Check the PCR products by agarose gel electrophoresis (sodium borate buffer 1×, 100 V, approximately 20 min) to confirm their correct size (~1,550 bp).
      v. Purify the PCR reaction(s) (PEG protocol by Travis Glenn).
      1) Transfer 18 μL of the respective PCR reaction(s) into a 1.5 mL tube and mix with 16 μL of PEG (20%) by constantly pipetting the solution up and down.
      2) Put it on the thermo shaker (37 °C, 800 rpm) for 15 min.
      3) Centrifuge at 15,000× *g* for 15 min at RT (21 °C).
      4) Discharge the supernatant (as it contains leftover polymerases, primer dimers, and unused dNTPs interfering with the subsequent sequencing reaction).
      5) Wash the pellet with 50 μL of ice-cold 80% ethanol (-20 °C).
      6) Centrifuge at 15,000× *g* for 2 min at RT (21 °C).
      7) Remove the supernatant.
      8) Repeat the centrifugation step and remove the supernatant completely.
      9) Incubate the tubes on the thermo shaker (37 °C and 800 rpm) with lid open until no trace (visible drops as well as smell) of ethanol is left.
      10) Add 18 μL of nuclease-free water.
      11) Pipette the nuclease-free water up and down several times (this ensures that the DNA is fully resuspended in the water).
      vi. Check the purified PCR products again with agarose gel electrophoresis (sodium borate buffer 1×, 100 V, approximately 20 min) (Figure 3).
      vii. Send for sequencing [using e.g., the Microsynth sequencing service (Balgach, Switzerland)].

## Data analysis

- **Wood decay rates**
  To ensure that the initial moisture content was uniform throughout all wood blocks and to calculate the estimated dry weight on day zero (dry m_0_), dry four moisture control specimen wood blocks at ~50 °C for 72 h after autoclaving and weighing (point B5). This can be done in parallel to the assay. Our specimen lost on average 0.6905 g (≙8.62%), hence 91.38% of the mass remained unchanged. Calculate dry m_0_ as: dry m_0_ = m d_0_ × 0.9138, with m d_0_ being the initial weight of the wood block after autoclaving (before exposure to microbes). Calculate mass loss (ML) difference as: m_T_ (g) = (dry m_0_ - m_1_), where m_T_ is the difference of ML after exposure to microorganisms, and m_1_ and dry m_0_ are the dry masses after and before degradation, respectively. The ML in % is the measure for the extent of fungal degradation [m_T_ (%) = (dry m_0_ ÷ m_1_) × 100]. Prepare a minimum of five replicates for each approach and of three replicates per control approach. Calculate average wood weight loss in % and standard deviation for each approach and illustrate e.g., in a boxplot. Conduct statistical analysis with *pairwise.t.test* function for pairwise comparison using *t*-tests with pooled SD (p-value adjustment method: holm and Bonferroni) in the psych package v2.2.5 of the R programming language (RStudio). Additionally, calculate single-factor variance analysis [ANOVA, function *aov()* in RStudio].
  A significantly higher weight loss in the co-cultivation approach than in the axenic fungal control is interpreted as a synergistic effect; lower weight losses are interpreted as antagonism.
- **Evaluation of PCR 16S rRNA placement**
  Check sequencing results for quality by evaluating the corresponding chromatograms. If 16S rRNA gene sequences of the applied microbes are available for comparison, compare initial sequences with the sequences of the re-isolated strains by aligning them. Alternatively, blast sequences of the 16S rRNA gene with the NCBI online tool. Limit the searching settings to 16S ribosomal RNA sequences (Bacteria and Archaea) and optimise for highly similar sequences (*megablast*). According to the results of the Blast search, assign the bacteria to genera and compare with the bacterial strains applied at the beginning. This allows to estimate if the bacteria survived the procedure.
*Note: Some mould spores may have survived and developed; we recommend recording affected plates/tins. We excluded these specimens from the calculations.*

## Notes

Prepare enough tins with pre-cultured fungus, as contaminations occur easily since some steps cannot be performed entirely sterile. Before starting the experiment, we recommend careful planning and listing of what should be prepared.

Mind the controls:

- Axenic fungal control: *S. lacrymans* with wood that is immersed with 0.85% NaCl solution.
- Wood sterile control: wood that is immersed with 0.85% NaCl solution, without microbes.
- Axenic bacterial control: wood that is immersed with bacterial suspension that was adjusted to 10^8^ CFU/mL without fungus.
- Minimum four moisture control specimen wood blocks (without them it is not possible to estimate the initial dry weight dry m_0_).
  It is also possible to sterilise the wood blocks via UV light, as this would preserve the initial state of the wood better than autoclaving. The disadvantage is that more moulds and other contaminants survive this procedure, which might cause problems in the experiment.
